# Supplementary material for: Superior Colliculus to VTA pathway controls orienting response and influences social interaction in mice
Source: Nat Commun. 2022 Feb 10;13:817. doi: 10.1038/s41467-022-28512-4 (PMC8831635; doi:10.1038/s41467-022-28512-4)
Supplement: Supplementary file 1 — Supplementary Information [file 41467_2022_28512_MOESM1_ESM.pdf]

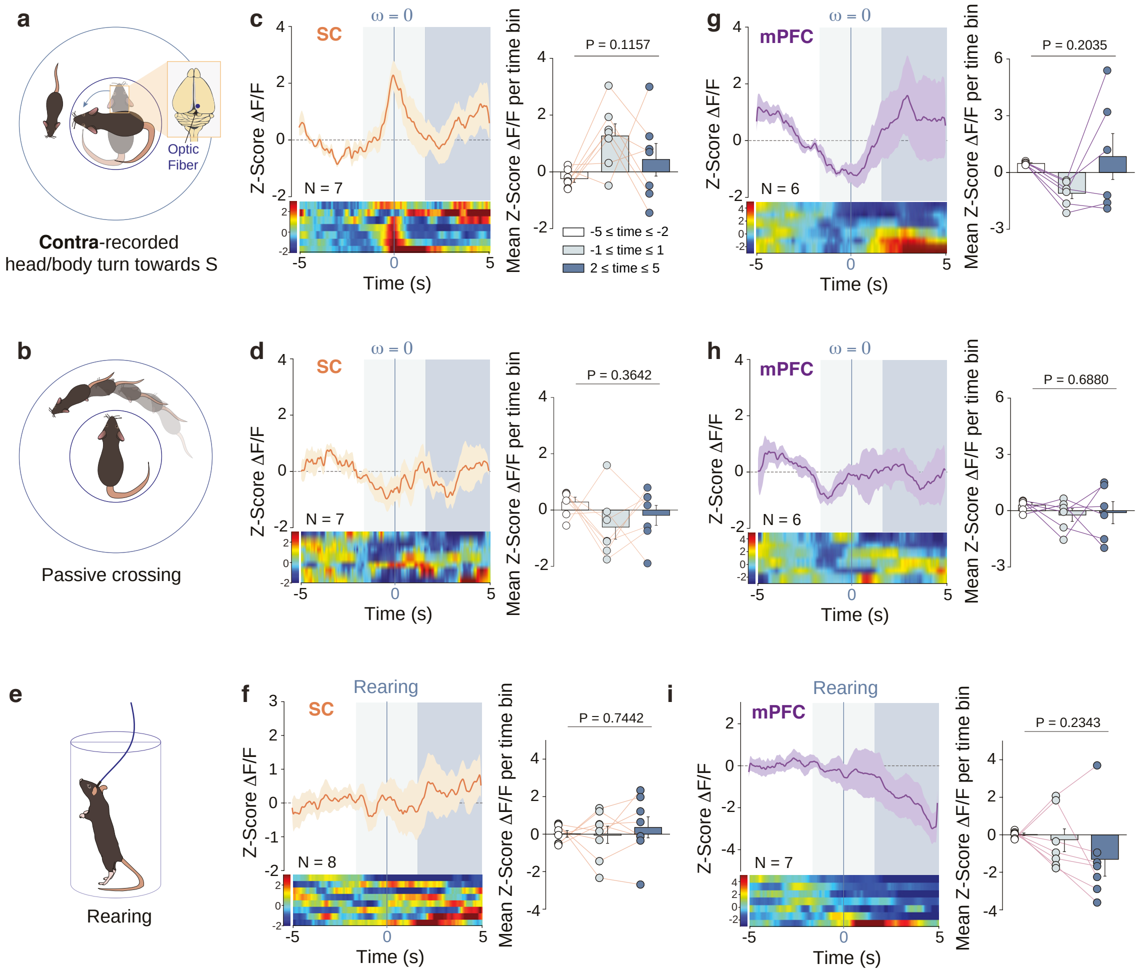

Sup. Figure 1 - Solié, Contestabile et all

Supplementary figure 1: Calcium activity of SC or mPFC to VTA projecting neurons during social orientation test.

(a, **b** and **e)** Time locked events observed during social orientation test. **(c)** Left panel: Peri-event time histogram (PETH) of normalized ΔF/F for SC-VTA projecting neurons, centered on contra-recorded orientation. Right panel: Mean ΔF/F (Z-score) before, during and after contra-recorded orientation towards social stimulus. RM one-way ANOVA (Events main effect: $F_{(2,6)}$ = 2.59, P = 0.1157). **(d)** Left panel: PETH of normalized ΔF/F for SC-VTA projecting neurons, centered on passive crossing. Right panel: Mean ΔF/F (Z-score) before, during and after passive crossing. RM one-way ANOVA (Events main effect: $F_{(2,6)}$ = 1.1002, P = 0.3642). **(f)** Left panel: PETH of normalized ΔF/F for SC-VTA projecting neurons, centered on rearing behaviour. Right panel: Mean calcium activity (Z-score) before, during and after rearing. RM one-way ANOVA (Events main effect: $F_{(2,7)}$ = 0.3017, P = 0.7442). **(g)** Left panel: PETH of normalized ΔF/F for mPFC-VTA projecting neurons, centered on contra-recorded orientation towards social stimulus. Right panel: Mean ΔF/F (Z-score) before, during and after contra-recorded orientation. RM one-way ANOVA (Events main effect: $F_{(2,5)}$ = 1.87, P = 0.2035). **(h)** Left panel: PETH of normalized ΔF/F for mPFC-VTA projecting neurons, centered on passive crossing. Right panel: Mean ΔF/F (Z-score) before, during and after passive crossing. RM one-way ANOVA (Events main effect: $F_{(2,5)}$ = 0.39, P = 0.6880). **(i)** Left panel: PETH of normalized ΔF/F for mPFC-VTA projecting neurons, centered on rearing behaviour. Right panel: Mean calcium activity (Z-score) before, during and after rearing. RM one-way ANOVA (Events main effect: $F_{(2,6)}$ = 1.6417, P = 0.2343). N indicates the number of mice. All the data are shown as the mean +/- s.e.m. as error bars or error bands. Source data are provided as a Source Data file.

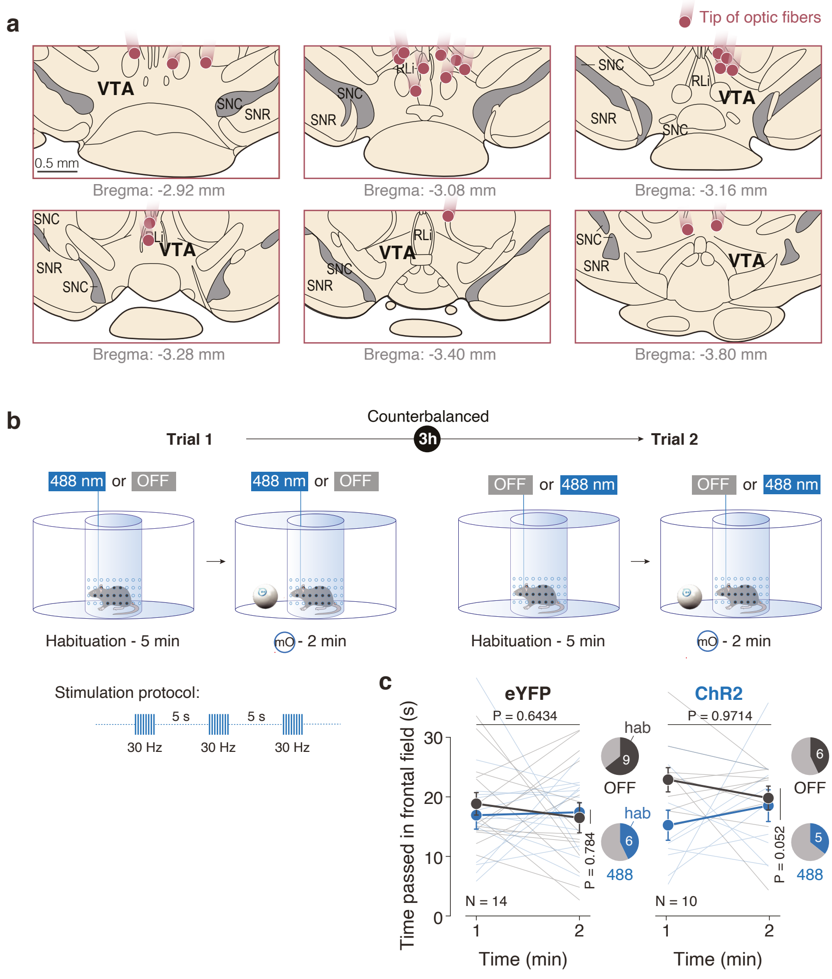

Sup. Figure 2 - Solié, Contestabile et all

Supplementary figure 2: Optogenetic manipulation of SC-VTA pathway alters orientation towards moving non-social stimuli.

(a) Examples of optic fibers' tips localization after post-hoc validation. **(b)** Top panel: Schema of the social orientation test. The eYFP- and ChR2-expressing mice oriented towards a moving ball (mO) during both stimulation conditions. Bottom panel: Stimulation protocol. 8 pulses of 488 nm light (30 Hz) were separated by 5s in the light ON condition. **(c)** Time passed with the social stimulus in the frontal field for the 1st and 2nd minute of the orienting test in light and no-light conditions. RM two-way ANOVA (eYFP$^{488\ nm}$: Light main effect: $F_{(1,13)} = 0.0782$, $P = 0.7842$; Time main effect: $F_{(1,13)} = 0.2247$, $P = 0.6434$; Light x Time Interaction: $F_{(1,13)} = 0.3365$, $P = 0.5718$. ChR2$^{488\ nm}$: Light main effect: $F_{(1,9)} = 4.984$, $P = 0.0525$; Time main effect: $F_{(1,9)} = 0.001$, $P = 0.9714$; Light x Time Interaction: $F_{(1,9)} = 3.252$, $P = 0.1049$). Pie charts represent the percentage of mice that decrease the orientation towards the moving ball between 1st and 2nd minute. N indicates the number of mice. All the data are shown as the mean +/- s.e.m. as error bars. Source data are provided as a Source Data file.

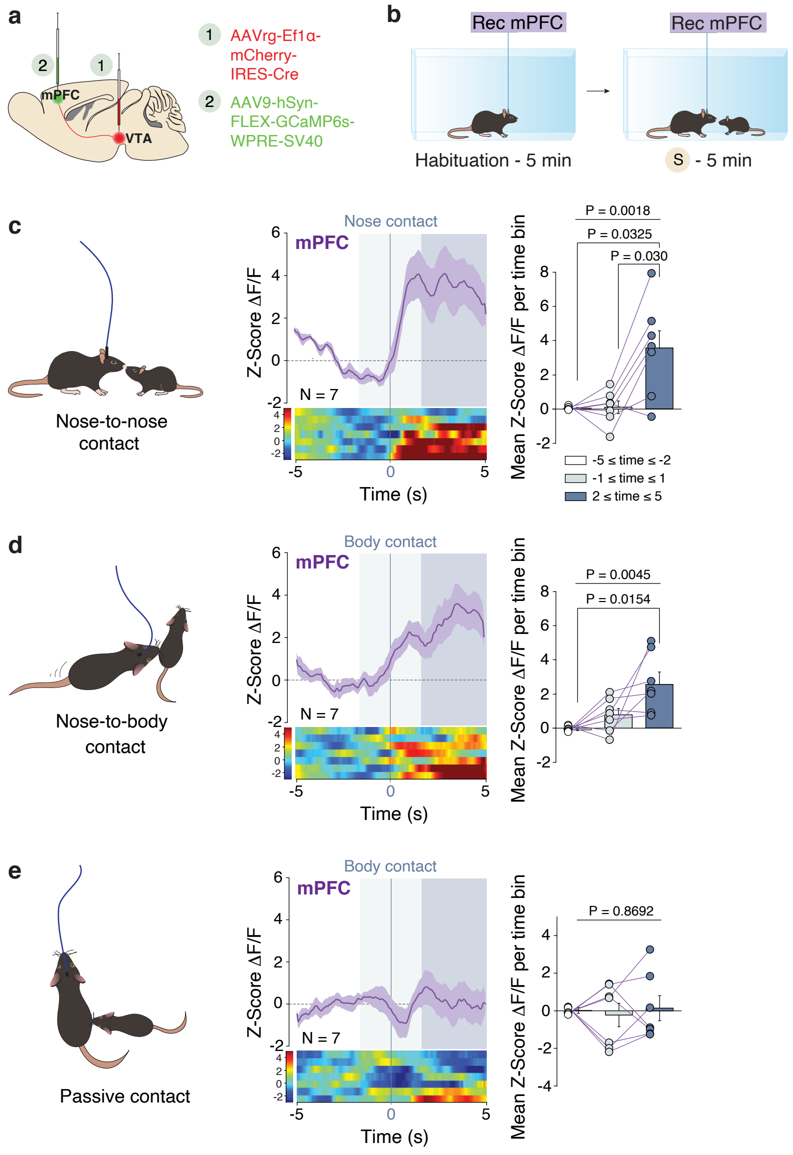

Sup. Figure 3 - Solié, Contestabile et all

Supplementary figure 3: Calcium activity of mPFC to VTA projecting neurons during free social interaction.

(a) Schema of injections of AAVrg-Ef1α-mCherry-IRES-Cre in the VTA and AAV9-hSyn-FLEX-GCaMP6s-WPRE-SV40 in the mPFC. **(b)** Schema of free social interaction test. **(c)** Left panel: PETH of normalized ΔF/F for mPFC-VTA projecting neurons, centered on nose-to-nose contacts. Right panel: Mean ΔF/F (Z-score) before, during and after nose-to-nose. RM one-way ANOVA (Events main effect: $F_{(2,6)} = 11.28$, $P = 0.0018$) followed by Bonferroni-Holm post-hoc test correction. **(d)** Left panel: PETH of normalized ΔF/F for mPFC-VTA projecting neurons, centered on nose-to-body contacts. Right panel: Mean ΔF/F (Z-score) before, during and after nose-to-body. RM one-way ANOVA (Events main effect: $F_{(2,6)} = 8.79$, $P = 0.0045$) followed by Bonferroni-Holm post-hoc test correction. **(e)** Left panel: PETH of normalized ΔF/F for mPFC-VTA projecting neurons, centered on passive contacts. Right panel: Mean ΔF/F (Z-score) before, during and after passive. RM one-way ANOVA (Events main effect: $F_{(2,6)} = 0.14$, $P = 0.8692$). N indicates the number of mice. All the data are shown as the mean +/- s.e.m. as error bars or error bands. Source data are provided as a Source Data file.

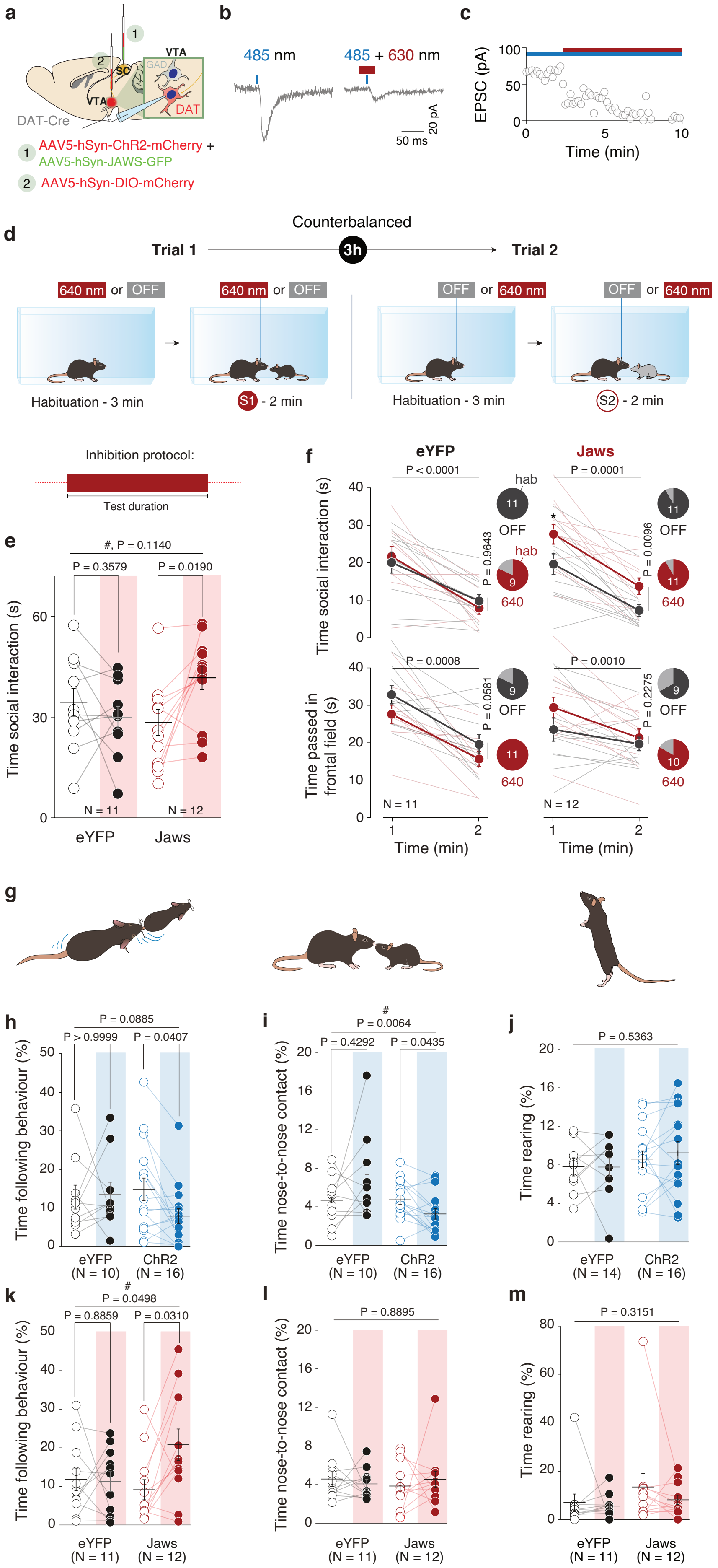

Sup. Figure 4 - Solié, Contestabile et all

Supplementary figure 4: Optogenetic inhibition of SC-VTA pathway alters social interaction and orientating responses.

(a) Schema of injections in the SC with AAV5-hSyn-ChR2-mCherry + AAV5-hSyn-JAWS-GFP and with AAV5-hSyn-DIO-mCherry in the VTA. **(b)** Example traces of evoked EPSCs after photostimulation (left) and photostimulation followed by photoinhibition (right). **(c)** Amplitude of evoked EPSCs in function of the time. Photostimulation is indicated in blue and photoinhibition in red. The graph shows an induced EPSC in the VTA DAT^+ neurons when the blue light only is shined. However, with contingent shining of blue and red lights, the current approach to 0 confirming a terminal inhibition from the SC onto VTA DAT^+ neurons. **(d)** Top panel: Schema of free social interaction. The eYFP- and Jaws-expressing mice freely interacted with two different unfamiliar mice under both stimulation conditions. Bottom panel. Inhibition protocols. Continuous inhibition was provoked with 640 nm light. **(e)** Time social interaction during the free social interaction test for eYFP- and Jaws-expressing mice in the SC. RM two-way ANOVA (Light main effect: $F_{(1,21)} = 2.7201$, $P = 0.1140$; Virus main effect: $F_{(1,21)} = 0.1985$, $P = 0.6605$; Light x Virus Interaction: : $F_{(1,21)} = 8.3378$, $P = 0.0088$) followed by Bonferroni-Holm post-hoc test correction. **(f)** Upper panels: time passed interacting with the social stimulus for the 1st and 2nd minute of the free social interaction test in light and no-light conditions. RM two-way ANOVA (eYFP: Light main effect: $F_{(1,10)} = 0.002$, $P = 0.9643$; Time main effect: $F_{(1,10)} = 49.49$, $P < 0.0001$; Light x Time Interaction: $F_{(1,10)} = 0.7046$, $P = 0.4209$. Jaws: Light main effect: $F_{(1,11)} = 9.803$, $P = 0.0096$; Time main effect: $F_{(1,11)} = 33.08$, $P = 0.0001$; Light x Time Interaction: $F_{(1,11)} = 0.1984$, $P = 0.6646$) followed by Bonferroni's multiple comparisons post-hoc test. Lower panels: time passed with the social stimulus in the frontal field for the 1st and 2nd minute of the free social interaction test in light and no-light conditions. RM two-way ANOVA (eYFP: Light main effect: $F_{(1,10)} = 4.576$, $P = 0.0581$; Time main effect: $F_{(1,10)} = 22.69$, $P = 0.0008$; Light x Time Interaction: $F_{(1,10)} = 0.1177$, $P = 0.7386$. Jaws: Light main effect: $F_{(1,11)} = 1.634$, $P = 0.2275$; Time main effect: $F_{(1,11)} = 19.89$, $P = 0.0010$; Light x Time Interaction: $F_{(1,11)} = 0.8598$, $P = 0.3737$) followed by Bonferroni's multiple comparisons post-hoc test. Pie charts represent the percentage of mouse that decreases the interaction/orientation between 1st and 2nd minute. **(g)** Schema of a mouse performing following behaviour (left), nose-to-nose (center) or

rearing (right). **(h)** Time of following behaviour for SC eYFP and ChR2-expressing mice under light-ON and light-OFF epochs. RM two-way ANOVA (Light main effect: $F_{(1,24)}$ = 3.1522, P = 0.0885; Virus main effect: $F_{(1,24)}$ = 0.3289, P = 0.5716; Interaction Light x Virus: $F_{(1,24)}$ = 2.7778, P = 0.1086) followed by Bonferroni-Holm post-hoc test correction. **(i)** Time of nose-to-nose contact for SC eYFP and ChR2-expressing mice under light-ON and light-OFF epochs. RM two-way ANOVA (Light main effect: $F_{(1,24)}$ = 0.0064, P = 0.9370; Virus main effect: $F_{(1,24)}$ = 4.3794, P = 0.0471; Interaction Light x Virus: $F_{(1,24)}$ = 6.4369, P = 0.0181) followed by Bonferroni-Holm post-hoc test correction. **(j)** Time of rearing behaviour during free social interaction test for SC eYFP and ChR2-expressing mice under light-ON and light-OFF epochs. RM two-way ANOVA (Light main effect: $F_{(1,28)}$ = 0.3921, P = 0.5363; Virus main effect: $F_{(1,28)}$ = 1.3238, P = 0.2596; Interaction Light x Virus: $F_{(1,28)}$ = 0.5985, P = 0.4456). **(k)** Time of following behavior for SC eYFP and Jaws-expressing mice under light-ON and light-OFF epochs. RM two-way ANOVA (Light main effect: $F_{(1,21)}$ = 4.3333, P = 0.0498; Virus main effect: $F_{(1,21)}$ = 0.9925, P = 0.3305; Interaction Light x Virus: $F_{(1,21)}$ = 4.7701, P = 0.0404) followed by Bonferroni-Holm post-hoc test correction. **(l)** Time of nose-to-nose contact for SC eYFP and Jaws-expressing mice under light-ON and light-OFF epochs. RM two-way ANOVA (Light main effect: $F_{(1,21)}$ = 0.0198, P = 0.8895; Virus main effect: $F_{(1,21)}$ = 0.0476, P = 0.8294; Interaction Light x Virus: $F_{(1,21)}$ = 0.5243, P = 0.4770). **(m)** Time of rearing behaviour during free social interaction test for SC eYFP and Jaws-expressing mice under light-ON and light-OFF epochs. RM two-way ANOVA (Light main effect: $F_{(1,21)}$ = 1.0594, P = 0.3151; Virus main effect: $F_{(1,21)}$ = 1.3808, P = 0.2531; Interaction Light x Virus: $F_{(1,21)}$ = 0.3329, P = 0.5701). # Indicates significant interaction. N indicates the number of mice. All the data are shown as the mean +/- s.e.m. as error bars. Source data are provided as a Source Data file.

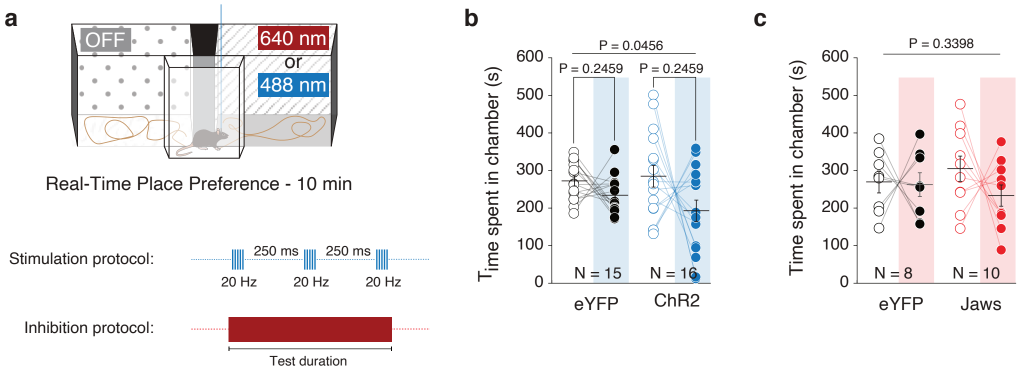

Sup. Figure 5 - Solié, Contestabile et all

Supplementary figure 5: SC-VTA pathway stimulation or inhibition does not induce place preference

(a) Schema of the real-time place preference set up. The optogenetic stimulation/inhibition is assigned to one chamber while the other one is not associated with any stimulation. The mice are free to explore the apparatus during 10 mins. **(b)** Time spent in the chamber associated with the photostimulation or not for eYFP and ChR2 mice. RM two-way ANOVA (Light main effect: $F_{(1,29)}$ = 4.3617, P = 0.0456; Virus main effect: $F_{(1,29)}$ = 5.2176, P = 0.0299; Light x Virus Interaction: $F_{(1,29)}$ = 0.7305, P = 0.3997) followed by Bonferroni-Holm post-hoc test correction. **(c)** Time spent in the chamber associated with the photoinhibition or not for eYFP and Jaws mice. RM two-way ANOVA (Light main effect: $F_{(1,16)}$ = 0.9683, P = 0.3398; Virus main effect: $F_{(1,16)}$ = 0.2699, P = 0.6105; Light x Virus Interaction: $F_{(1,16)}$ = 0.5421, P = 0.4722). N indicates the number of mice. All the data are shown as the mean +/- s.e.m. as error bars. Source data are provided as a Source Data file.

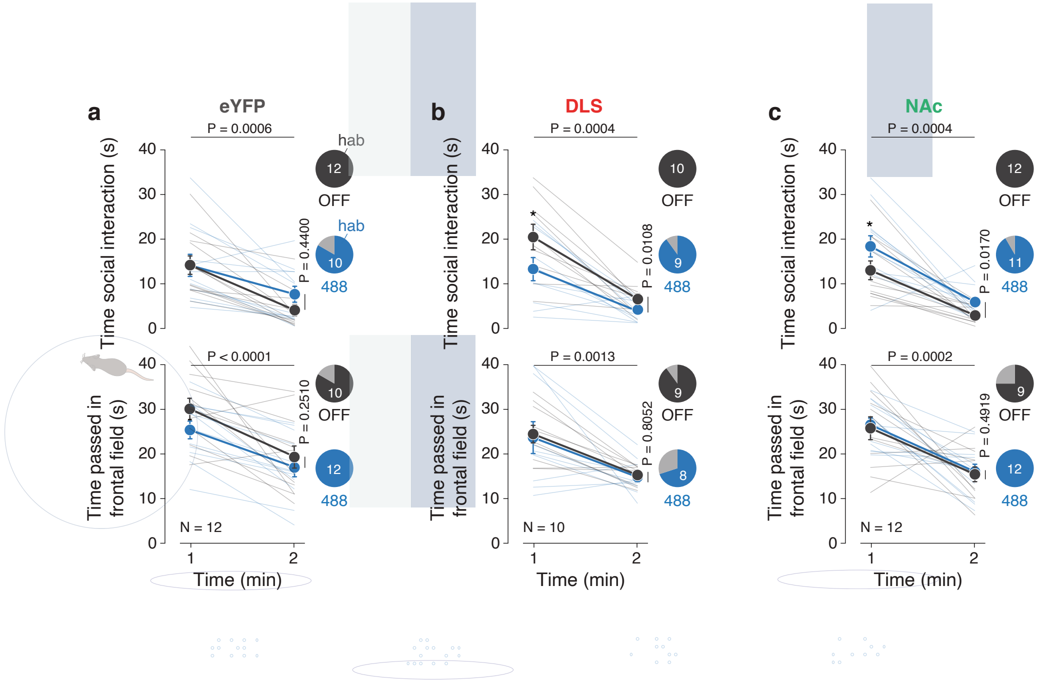

Sup. Figure 6 - Solié, Contestabile et all

Supplementary figure 6: Optogenetic stimulation of VTA-DLS or VTA-NAc pathways do not perturb head orientation towards conspecific.

(**a**, **b** and **c)** Upper panels: time passed interacting with the social stimulus for the 1st and 2nd minute of the free social interaction test in light and no-light conditions. RM two-way ANOVA (eYFP: Light main effect: $F_{(1,11)} = 0.6419$, $P = 0.4400$; Time main effect: $F_{(1,11)} = 22.40$, $P = 0.0006$; Light x Time Interaction: $F_{(1,11)} = 2.799$, $P = 0.1225$. $ChR2^{DLS}$: Light main effect: $F_{(1,9)} = 10.26$, $P = 0.0108$; Time main effect: $F_{(1,9)} = 29.19$, $P = 0.0004$; Light x Time Interaction: $F_{(1,9)} = 2.259$, $P = 0.1671$. $ChR2^{NAc}$: Light main effect: $F_{(1,11)} = 7.882$, $P = 0.0170$; Time main effect: $F_{(1,11)} = 24.51$, $P = 0.0004$; Light x Time Interaction: $F_{(1,11)} = 1.261$, $P = 0.2854$) followed by Bonferroni's multiple comparisons post-hoc test. Lower panels: time passed with the social stimulus in the frontal field for the 1st and 2nd minute of the free social interaction test in light and no-light conditions. RM two-way ANOVA (eYFP: Light main effect: $F_{(1,11)} = 1.469$, P = 2510; Time main effect: $F_{(1,11)} = 37.84$, $P < 0.0001$; Light x Time Interaction: $F_{(1,11)} = 0.3936$, $P = 0.5432$. $ChR2^{DLS}$: Light main effect: $F_{(1,9)} = 0.0645$, $P = 0.8052$; Time main effect: $F_{(1,9)} = 21.34$, $P = 0.0013$; Light x Time Interaction: $F_{(1,9)} = 0.006$, $P = 0.9388$. $ChR2^{NAc}$: Light main effect: $F_{(1,11)} = 0.5055$, $P = 0.4919$; Time main effect: $F_{(1,11)} = 29.27$, $P = 0.0002$; Light x Time Interaction: $F_{(1,11)} = 0.0011$, $P = 0.9742$) followed by Bonferroni's multiple comparisons post-hoc test. Pie charts represent the percentage of mouse that decreases the interaction/orientation between 1st and 2nd minute. N indicates the number of mice. All the data are shown as the mean +/- s.e.m. as error bars. Source data are provided as a Source Data file.
