# Supplementary material for: Superior Colliculus to VTA pathway controls orienting response and influences social interaction in mice
Source: Nat Commun. 2022 Feb 10;13:817. doi: 10.1038/s41467-022-28512-4 (PMC8831635; doi:10.1038/s41467-022-28512-4)
Supplement: Supplementary file 3 — Reporting Summary [file 41467_2022_28512_MOESM3_ESM.pdf]

nature portfolio

Corresponding author(s): Camilla Bellone

Last updated by author(s): Jan 5, 2022

Reporting Summary

Nature Portfolio wishes to improve the reproducibility of the work that we publish. This form provides structure for consistency and transparency in reporting. For further information on Nature Portfolio policies, see our Editorial Policies and the Editorial Policy Checklist.

Statistics

For all statistical analyses, confirm that the following items are present in the figure legend, table legend, main text, or Methods section.

n/a	Confirmed	
☐	☒	The exact sample size (*n*) for each experimental group/condition, given as a discrete number and unit of measurement
☐	☒	A statement on whether measurements were taken from distinct samples or whether the same sample was measured repeatedly
☐	☒	The statistical test(s) used AND whether they are one- or two-sided<br>*Only common tests should be described solely by name; describe more complex techniques in the Methods section.*
☒	☐	A description of all covariates tested
☐	☒	A description of any assumptions or corrections, such as tests of normality and adjustment for multiple comparisons
☐	☒	A full description of the statistical parameters including central tendency (e.g. means) or other basic estimates (e.g. regression coefficient) AND variation (e.g. standard deviation) or associated estimates of uncertainty (e.g. confidence intervals)
☐	☒	For null hypothesis testing, the test statistic (e.g. *F*, *t*, *r*) with confidence intervals, effect sizes, degrees of freedom and *P* value noted<br>*Give P values as exact values whenever suitable.*
☒	☐	For Bayesian analysis, information on the choice of priors and Markov chain Monte Carlo settings
☒	☐	For hierarchical and complex designs, identification of the appropriate level for tests and full reporting of outcomes
☒	☐	Estimates of effect sizes (e.g. Cohen's *d*, Pearson's *r*), indicating how they were calculated

Our web collection on statistics for biologists contains articles on many of the points above.

Software and code

Policy information about availability of computer code

Data collection	Ethovision XT 16, Noldus<br>Imetronic video acquisition for optogenetic, Pessac, France<br>Doric System fiber photometry, Doric Lenses inc.<br>Microscopes Zeiss Software LSM700 and LSM800<br>Axioscan.Z1 Scanner Software
Data analysis	MATLAB_R2019b (The Mathwork)<br>Ethovision XT 16, Noldus<br>Imetronic video acquisition, Pessac, France<br>GraphPad Prism 7 (San Diego, CA, USA)<br>RStudio 1.3<br>MetaMorph Microscopy Automation and Image Analysis Software, Molecular Devices

For manuscripts utilizing custom algorithms or software that are central to the research but not yet described in published literature, software must be made available to editors and reviewers. We strongly encourage code deposition in a community repository (e.g. GitHub). See the Nature Portfolio guidelines for submitting code & software for further information.

Data

Policy information about availability of data

All manuscripts must include a data availability statement. This statement should provide the following information, where applicable:

- Accession codes, unique identifiers, or web links for publicly available datasets
- A description of any restrictions on data availability
- For clinical datasets or third party data, please ensure that the statement adheres to our policy

Original data used in the present study are available in the following link: XXXXXXXXXXXXX. Further data supporting the findings are available upon request.
Find all the Source Data files attached to the manuscript. Each data file is provided for each figure and supplementary figure.

Field-specific reporting

Please select the one below that is the best fit for your research. If you are not sure, read the appropriate sections before making your selection.

☒ Life sciences ☐ Behavioural & social sciences ☐ Ecological, evolutionary & environmental sciences

For a reference copy of the document with all sections, see nature.com/documents/nr-reporting-summary-flat.pdf

Life sciences study design

All studies must disclose on these points even when the disclosure is negative.

Sample size	No statistical methods were used to predetermine the number of animals and cells, but suitable sample sizes were estimated based on previous experience and are similar to those generally employed in the field.<br>Zhou, Z. et al. A VTA GABAergic Neural Circuit Mediates Visually Evoked Innate Defensive Responses. Neuron 103, 473-488.e6 (2019).<br>Nuno-Perez, A. et al. Stress undermines reward-guided cognitive performance through synaptic depression in the lateral habenula. Neuron 109, 947-956.e5 (2021).
Data exclusions	Statistical outliers were identified by using the criterion Mean(Value) ± 3 × Std(value). However, in our study, no outliers have been identified using this criterion.
Replication	The behavioral aspect of the free social interaction has been replicated in several cohorts in this manuscript, 8 times to obtain all the groups: for the fiber photometry in the Sup. Colliculus (SC) and in the mPFC, for the optogenetic with eYFP, ChR2 and Jaws and for the optogenetic with eYFP and ChR2 targeting VTA - DLS and VTA - NAc pathways.<br>The social orientation task has also been replicated in several batches, 8 times to obtain all the groups: for the fiber photometry in the SC for social and moving object stimuli and in the mPFC, for the optogenetic with eYFP, ChR2 and Jaws for social stimulus and with eYFP and ChR2 for moving object stimulus.<br>The Open field test and the real time place preference are tasks widely used in behavioural neuroscience field. The reproducibility of these tests consisted in preventing any escaping of the arena from the mice.
Randomization	All the experimental mice used in the study were wild-type (WT), GAD2-Cre or DAT-Cre male transgenic mice. All the stimuli mice were WT male juvenile mice. The stimuli mice were randomly associated with experimental mice for the different tasks.<br>For behavioural and fiberphotometry tracking, we used Ethovision to perform, as much as possible, non-biased automatic analyses in coupling calcium recording and behaviour.<br>For manual scoring of nose-to-nose, following, rearing and grooming behaviours, the experimenters were assigned randomly to the scoring.
Blinding	All the experimenters were blinded at group allocation during data collection and analysis.

Reporting for specific materials, systems and methods

We require information from authors about some types of materials, experimental systems and methods used in many studies. Here, indicate whether each material, system or method listed is relevant to your study. If you are not sure if a list item applies to your research, read the appropriate section before selecting a response.

Materials & experimental systems

n/a	Involved in the study	
☐	☒	Antibodies
☒	☐	Eukaryotic cell lines
☒	☐	Palaeontology and archaeology
☐	☒	Animals and other organisms
☒	☐	Human research participants
☒	☐	Clinical data
☒	☐	Dual use research of concern

Methods

n/a	Involved in the study	
☒	☐	ChIP-seq
☒	☐	Flow cytometry
☒	☐	MRI-based neuroimaging

Antibodies

Antibodies used	In this study, the following primary antibody were used: rabbit polyclonal anti-Tyrosine Hydroxylase (1/500 dilution, abcam, ab6211); Rabbit polyclonal anti-mCherry (1/200 dilution, abcam ab167453); Mouse monoclonal anti-CaMKII alpha (1/100 dilution, ThermoFisher, MA1_048, RRID: AB_325403).<br>The following secondary antibody were used at 1/500 dilution: donkey anti-rabbit IgG H&L 555 (Alexa Fluor) (ThermoFisher, A32794, RRID: AB_2762834), donkey anti-mouse IgG H&L 488 (Alexa Fluor) (ThermoFisher, R37114, RRID: AB_2556542); donkey anti-rabbit IgG H&L 647 (Alexa Fluor) (ThermoFisher, A32795, RRID: AB_2762835).<br>To mount the slices the following antibodies was used to tag the nucleus of cells: Fluoroshield mounting with DAPI (abcam ab104139)
Validation	According to the manufacter's website (abcam), ab6211 has been referenced in 41 publications. Find all the publications on this link: https://www.abcam.com/tyrosine-hydroxylase-antibody-ab6211.html<br><br>According to the manufacter's website (abcam), ab167453 has been referenced in 204 publications. Find all the publications on this link: https://www.abcam.com/mcherry-antibody-ab167453.html<br><br>According to the manufacter's website (ThermoFisher), MA1_048 has been referenced in 73 publications. Find all the publications on this link: https://www.thermofisher.com/antibody/product/CaMKII-alpha-Antibody-clone-6G9-Monoclonal/MA1-048<br><br>According to the manufacter's website Thermofisher, R37114 has been referenced in 26 publications: https://www.thermofisher.com/antibody/product/Donkey-anti-Mouse-IgG-H-L-Secondary-Antibody-Polyclonal/R37114<br><br>According to the manufacter's website Thermofisher, A32794 has been referenced in 1 publication: https://www.thermofisher.com/antibody/product/Donkey-anti-Rabbit-IgG-H-L-Highly-Cross-Adsorbed-Secondary-Antibody-Polyclonal/A32794<br><br>According to the manufacter's website Thermofisher, A32795 has been referenced in 3 publications: https://www.thermofisher.com/antibody/product/Donkey-anti-Rabbit-IgG-H-L-Highly-Cross-Adsorbed-Secondary-Antibody-Polyclonal/A32795<br><br>According to the manufacter's website (Abcam), ab104139 has been referenced in 117 publications: https://www.abcam.com/mounting-medium-with-dapi-aqueous-fluoroshield-ab104139.html

Animals and other organisms

Policy information about studies involving animals; ARRIVE guidelines recommended for reporting animal research

Laboratory animals	The study was conducted using wild type (WT) and transgenic mice with C57BL/6J background. WT mice were obtained from Charles River and used as experimental and stimuli mice. For DA neuron-specific manipulations and labelling, DAT-iresCre (Slc6a3tm1.1(cre)Bkmn/J) were employed. For GABA neurons labelling, GAD2-iresCre (Gad2tm2(creZjh)/J called GAD65-Cre) were used. Only males' animals were used for all the experiments conducted. Mice were housed in groups (weaning at P21 – P23) under a 12 hours light – dark cycle (7:00 a.m.–7:00 p.m.). All physiology and behaviour experiments in experimental mice were performed when they reached the age of 8 weeks, while mice used as stimuli were around P25. All the expriments occured during the light cycle.
Wild animals	No wild animals were used in this study
Field-collected samples	No field-collected samples were used in this study.
Ethics oversight	All the procedures performed at UNIGE complied with the Swiss National Institutional Guidelines on Animal Experimentation and were approved by the respective Swiss Cantonal Veterinary Office Committees for Animal Experimentation.

Note that full information on the approval of the study protocol must also be provided in the manuscript.
